# Minimally Invasive Partial vs. Total Adrenalectomy for the Treatment of Unilateral Primary Aldosteronism: A Systematic Review and Meta-Analysis

**DOI:** 10.3390/jcm11051263

**Published:** 2022-02-25

**Authors:** Rocco Simone Flammia, Umberto Anceschi, Antonio Tufano, Eugenio Bologna, Flavia Proietti, Alfredo Maria Bove, Leonardo Misuraca, Riccardo Mastroianni, Giuseppe Tirone, Alessandro Carrara, Lorenzo Luciani, Tommaso Cai, Costantino Leonardo, Giuseppe Simone

**Affiliations:** 1 Urology Unit, Department of Maternal-Child and Urological Sciences, “Sapienza” University of Rome, Viale del Policlinico 155, 00161 Rome, Italy; roccosimone92@gmail.com (R.S.F.); antonio.tufano@gmail.com (A.T.); eugenio.bologna@gmail.com (E.B.); flavia.proietti@gmail.com (F.P.); alfredo.bove@ifo.gov.it (A.M.B.); leonardo.misuraca@gmail.com (L.M.); riccardomastroianniroma@gmail.com (R.M.); costantino.leonardo@gmail.com (C.L.); 2 Department of Urology, IRCCS “Regina Elena” National Cancer Institute, Via Elio Chianesi 53, 00144 Rome, Italy; puldet@gmail.com; 3 Department of General Surgery, Santa Chiara Regional Hospital, Azienda Sanitaria per i Servizi Sanitari (APSS), Largo Medaglie d’Oro 9, 38122 Trento, Italy; giuseppe.tirone@apss.tn.it; 4 Department of General Surgery, Santa Maria del Carmine Hospital, Azienda Sanitaria per i Servizi Sanitari (APSS), Corso Verona 4, 38068 Rovereto, Italy; alessandro.carrara@apss.tn.it; 5 Department of Urology, Santa Chiara Regional Hospital, Azienda Sanitaria per i Servizi Sanitari (APSS), Largo Medaglie d’Oro 9, 38122 Trento, Italy; lorenzo.luciani@apss.tn.it (L.L.); ktommy@libero.it (T.C.)

**Keywords:** unilateral primary aldosteronism, Conn’s syndrome, partial adrenalectomy, total adrenalectomy, PASO

## Abstract

Background: This systematic review and metanalysis was conducted to assess differences between perioperative and functional outcomes in patients undergoing minimally-invasive partial (mi-PA) and total adrenalectomy (mi-TA) for unilateral primary aldosteronism (uPHA). Material and Methods: Multiple scientific databases (PUBMED, Web of Science, and Cochrane Library) were searched up to November 2021 for surgical series comparing mi-PA vs. mi-TA for uPHA according to the PRISMA statement. Primary outcomes of interest were perioperative and functional outcomes. Results: Overall, a total of 802 patients from six eligible studies were identified, with mi-PA and mi-TA performed in 40.4% (*n* = 324) and 59.6% (*n* = 478) of cases, respectively. No differences were recorded between the two groups according to number of transfusions, EBL and Clavien–Dindo complications ≥2. Similarly, no differences in clinical success, persistence of postoperative hypokalemia and improvement in HTN were reported between mi-PA and mi-TA. Conclusions: In a uPHA setting, mi-PA and mi-TA provide comparable perioperative and functional outcomes despite the use of mi-PA remains limited to patients with small adenoma size, or hereditary/bilateral disease. Due to limited use of standardized reporting criteria in most of current series, the quest for a superiority of mi-PA over mi-TA in the treatment of uPHA still remains open.

## 1. Introduction

Hypertension (HTN) represents a major cardiovascular risk factor [1]. Although many patients may suffer of essential HTN (eHTN), up to 15% are affected by undetected primary hyperaldosteronism (PHA), which represents the most common form of secondary HTN [2]. In recent years, the introduction of plasma aldosterone-to-plasma renin activity ratio (ARR), as a screening test in selected patients with HTN, has led to a 5–15-fold increase in the diagnosis of PHA, resulting in an estimated prevalence of 4% in the general population [3,4,5]. To parity of blood pressure elevation, patients affected by PHA have higher morbidity and mortality than patients with eHTN [6]. 

Clinical subtypes of PHA include unilateral, aldosterone producing adenoma (uPHA), unilateral adrenal hyperplasia (uAH), and bilateral adrenal hyperplasia (bAH) [7]. According to urological guidelines, minimally-invasive total adrenalectomy (mi-TA) represents the gold standard treatment for unilateral subtypes of PHA. Conversely, mineral corticoid receptor antagonists (MRAs) are recommended for medical treatment of bAH [8,9]. Nowadays, several authors have reported feasibility of adrenal-sparing techniques for uPHA with satisfactory outcomes for partial adrenalectomy (PA) vs. standard total adrenalectomy (TA) [10,11].

PA was originally described for the treatment of hereditary and sporadic bilateral tumors in order to reduce the risk of Addisonian crisis and to obviate the need for steroid replacement [12]. Nonetheless, promising results from the first randomized trial comparing PA versus TA [13] plus the wider adoption of minimally-invasive techniques have supported an increasing trend toward PA in the last two decades [14,15]. Although the feasibility of an adrenal-sparing approach has been reported by previous series, indications to PA remain limited [16,17]. In this context, the current literature has been recently subjected to meta-analysis, but not exclusively in the setting of minimally-invasive surgery [18,19,20]. 

To overcome these limitations, we sought to perform a systematic review and metanalysis of all available data from mi-PA and mi-TA series to evaluate differences in perioperative and functional outcomes between these surgical approaches for the management of uPHA. 

## 2. Material and Methods

A systematic search was conducted to find relevant studies from PubMed, Web of Science, Cochrane Central Register of Controlled Trials—CENTRAL (in the Cochrane Library, Issue 1, 2011), and Clinicaltrials.gov (accessed on 20 December 2021) according to the PRISMA statement [21]. The research was restricted to English language studies, published between January 2005 and November 2021. We used the Population, Intervention, Comparator, and Outcome (PICO) approach to define study eligibility [22,23], as following:

- Population: patients affected by uPHA with indication to surgical treatment;
- Intervention: minimally-invasive partial adrenalectomy (mi-PA);
- Comparator: minimally-invasive total adrenalectomy (mi-TA);
- Outcomes: perioperative and functional results.

### 2.1. Search Strategy

The searching strategy was designed using both free text and mesh terms. We used the following keywords: partial adrenalectomy, adrenal-sparing surgery, (Adrenal Cortex Neoplasms [MeSH Terms] AND partial adrenalectomy), (Adrenocortical Adenoma [MeSH Terms] AND partial adrenalectomy), (Hyperaldosteronism [Mesh Terms] AND partial adrenalectomy), (Laparoscopy [Mesh Terms] AND partial adrenalectomy), (Minimally Invasive Surgical Procedures [Mesh Terms] AND partial adrenalectomy), and (Robotic Surgical Procedures [Mesh Terms] AND partial adrenalectomy). 

### 2.2. Selection of Eligible Studies and Data Extraction 

**Figure 1:**
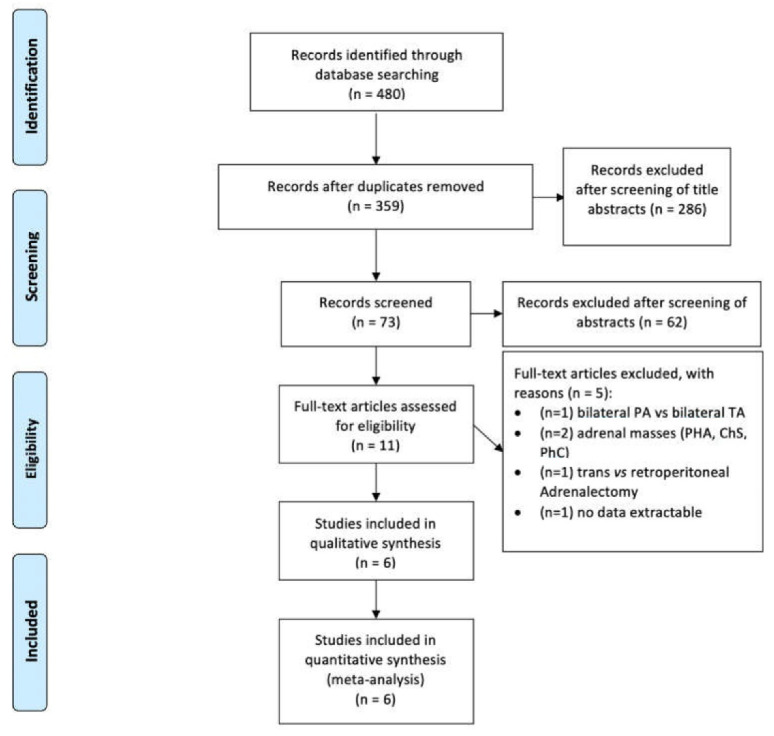
PRISMA flow chart.

References of selected papers were retrieved for preliminary inclusion. The full-text screening and data extraction were subsequently performed by two independent reviewers (A.T. and R.S.F.). Discrepancy was resolved by internal discussion or by supervision of an independent arbiter (U.A.). Article selection was performed according to the PRISMA flow-chart (Figure 1). Studies comparing mi-TA with mi-PA were identified (*n* = 6). 

### 2.3. Data Quality Assessment 

Quality of the included studies was assessed by the Risk Of Bias In Non-randomized Studies of Interventions (ROBINS-I) tool [24] and Risk-of-Bias tool for randomized trials (RoB 2) [25] as suggested by Cochrane handbook (Supplementary Figure S1). 

Moreover, all studies were classified according to the grade of evidence for therapy/prevention/etiology/harm studies by Phillips and Sackett [26] with the following order: meta-analyses of randomized clinical trials (RCTs) representing the highest evidence (level 1a), adequately sampled single RCT (level 1b), systematic review of cohort studies (level 2a), and low-quality RCT or observational studies (level 2b), surgical series (level 4), and expert opinion (level 5).

The following variables were extracted from each study:

- Baseline characteristics: age, BMI, gender, ASA score, HTN duration, number of antihypertensive medications, preoperative systolic blood pressure (SBP), diastolic blood pressure (DBP), serum Aldosterone (sA), serum Renin Activity (sRA), serum Aldosterone Renin Activity Ratio (ARR), and serum Potassium (sP);
- Perioperative outcomes: surgical approach (retro/transperitoneal), minimally-invasive technique (laparoscopic/robot-assisted), tumor size, operating time (OR), estimated blood loss (EBL), length of hospital stay (LOS), intraoperative transfusions rate, postoperative complications, and histopathological diagnosis;
- Functional outcomes: clinical and biochemical success according to standardized PASO criteria [27] (complete, partial, absent), postoperative SBP, DBP, postoperative hypokalemia, sA, sRA, sP, and recurrence rate.

### 2.4. Data Analysis

Cumulative meta-analysis of comparative studies was performed as follows. For continuous variables, a Mantel–Haenszel Chi-square test was used and expressed as the mean difference (MD) with 95% confidence interval (CI). For dichotomous variables, an inverse variance was used and expressed as odds ratio (OR) with 95% CI. In both cases *p*-value < 0.05 was considered significant. Both MD and OR were calculated comparing PA (experimental group) versus TA (control group). In case of positive outcomes, such as complete, partial clinical success, and improved hypertension; an OR > 1 indicated an advantage in the experimental arm (mi-PA). Heterogeneity was analyzed using a Chi-square test on n-of−1 degree of freedom, with a *p*-value < 0.05 used for statistical significance and with the I^2^ test for assessment of heterogeneity [28]. I^2^ values of 25%, 50%, and 90% corresponded to low, medium, and high levels of heterogeneity, respectively. Random effects and fixed effects were used in case of presence or absence of heterogeneity, respectively. RevMan (Review Manager) 5.4 was used for statistical analysis. Due to intrinsic limitations of this software, analysis of continuous variables was possible only when data were presented as mean and standard deviation (SD). Since some studies reported continuous variables in “median” and “interquartile range” or “min/max” range, we used a validated mathematical method (McGrath) to estimate “mean” and “SD” [29].

## 3. Results

### 3.1. Study Selection

The original search strategy retrieved 480 studies, published between January 2005 and November 2021, from which 469 were excluded based on titles and abstracts review. After evaluation of full manuscripts (11), only six studies comparing mi-PA to mi-TA were considered (Table 1).

Considering excluded studies, Liao et al. compared only bilateral PA versus bilateral TA [30], while Chen et al. focused on differences between retroperitoneal and transperitoneal approach [31]. Furthermore, two studies compared mi-PA and mi-TA in adrenal masses associated to different etiologies (Conn’s syndrome, Cushing’s syndrome, and Pheochromocytoma) [32,33]. Finally, Waltz et al. reported their experience on minimally-invasive adrenalectomy for PHA, without providing a comparison between PA vs. TA [14]. Conversely, among eligible studies, we identified only one randomized controlled trial (level of evidence 1b) [34]; one prospective non-randomized study (level of evidence: 2b) [35]; and four retrospective studies (level of evidence: 3b [10,11,36] or 4 [37]), respectively. 

Overall, 802 patients were enrolled in these studies, with mi-PA and mi-TA performed in 324 (40.4%) and 478 (59.6%) patients, respectively. Among cohorts, 778 (97%) patients were treated by laparoscopic adrenalectomy while 24 (3%) with a robotic-assisted technique. Demographic and surgical characteristics of patients were analyzed including age, gender, BMI, ASA, tumor size, side, surgical approach, HTN duration, preoperative anti-HTN drugs, SBP, DBP, sP, sA, and sRA (Table 2). 

We did not find any significant difference between the two cohorts except for BMI (MD: 0.92; 95% CI: 0.24, 1.61; kg/mt^2^; *p* = 0.008), DBP (MD: −4.87; 95% CI: −7.96, −1.79; mmHg; *p* = 0.002;), and HTN duration (MD: 0.30; 95% CI: 0.58–0.01; years; *p* = 0.04) (Supplementary Figure S2).

### 3.2. Perioperative Outcomes

**Figure 2:**
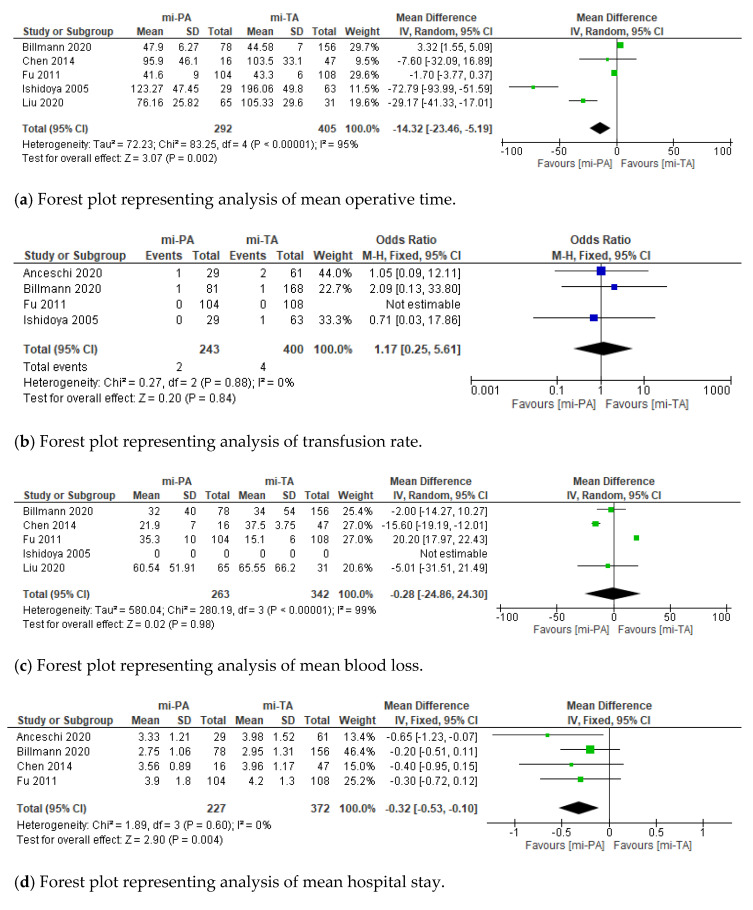

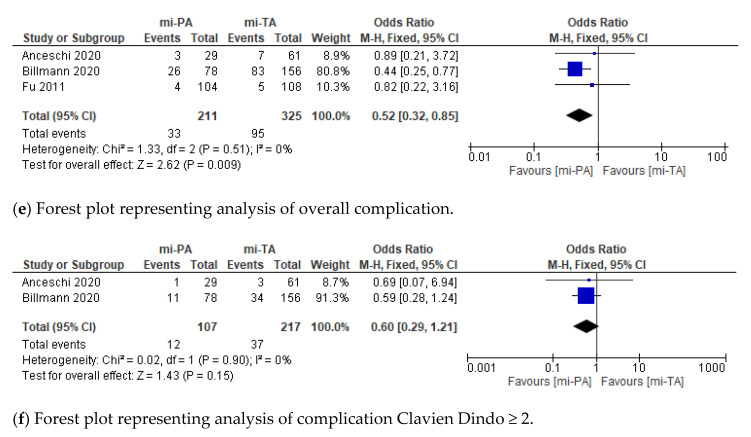
Cumulative analysis of eligible studies comparing mi-PA vs. mi-TA in terms of (**a**) operative time, (**b**) transfusion rate, (**c**) blood loss, (**d**) hospital stay, (**e**) overall complication, and (**f**) Clavien–Dindo ≥ 2. IV = inverse variance; SD = standard deviation; CI = confidence interval; M–H = Mantel–Haenszel. Each studies is represented by a square incorporating confidence intervals represented by horizontal lines. The area of each square is proportional to the study’s weight in the meta-analysis. The meta-analysed measure of effect is plotted as a diamond with lateral points indicating confidence intervals.

With regard to perioperative outcomes, we recorded a significant statistical difference between mi-PA and mi-TA according to mean operative time (MD: 14.32; 95% CI: −23.46, 5.19; minutes; *p* = 0.002), hospital stay (MD: −0.32; 95% CI: −0.53, −0.10; days; *p* = 0.004), and overall complication rate (OR: 0.52; 95% CI: 0.32, 0.85; *p* = 0.009), respectively. Conversely, no difference was found between groups in terms of number of transfusions, EBL, and Clavien–Dindo complications ≥ 2 (Figure 2). At final pathological report, no diagnosis of malignant tumor and different rate of solitary adenoma vs. hyperplasia was observed between groups (90% vs. 10% in mi-PA and 81% vs. 19% in mi-TA; OR 1.55; 95% CI: 1.01, 2.38; *p* = 0.04).

### 3.3. Functional Outcomes

**Figure 3:**
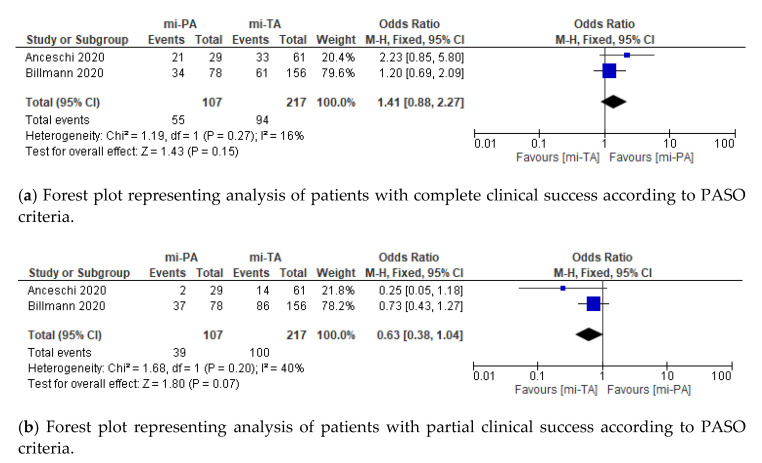

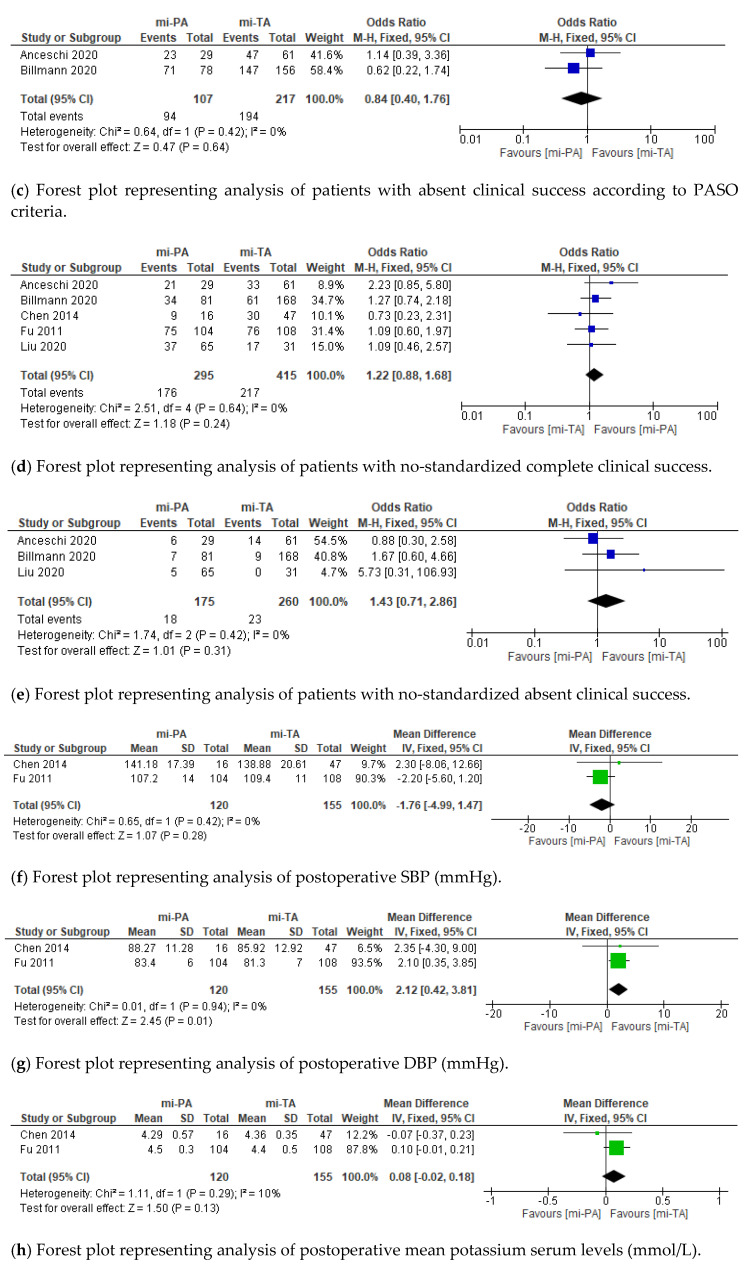

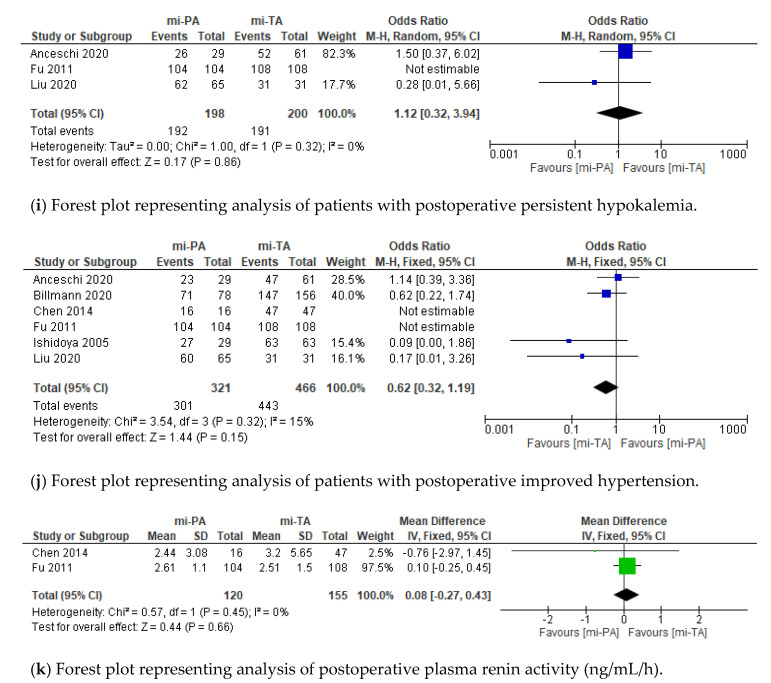
Cumulative analysis of eligible studies comparing mi-PA vs. mi-TA in terms of (**a**) complete clinical success according to PASO criteria, (**b**) partial clinical success according to PASO criteria, (**c**) absent clinical success according to PASO criteria, (**d**) no-standardized complete clinical success, (**e**) no-standardized absent clinical success, (**f**) postoperative SBP, (**g**) postoperative DBP, (**h**) postoperative mean potassium serum levels, (**i**) postoperative persistent hypokalemia, (**j**) postoperative improved hypertension, and (**k**) postoperative plasma renin activity. IV = inverse variance; SD = standard deviation; CI = confidence interval; M-H = Mantel-Haenszel. Each studies is represented by a square incorporating confidence intervals represented by horizontal lines. The area of each square is proportional to the study’s weight in the meta-analysis. The meta-analysed measure of effect is plotted as a diamond with lateral points indicating confidence intervals.

According to PASO criteria, complete, partial, and absent clinical success analyzed over 324 patients from two series showed no differences [10,36]. Moreover, similar results were found when considering no-standardized complete and absent clinical success over five studies for a total of 720 patients included [10,11,34,35,36]. No differences in persistence of postoperative hypokalemia and improvement in HTN were reported between mi-PA and mi-TA. Only two recurrences, confirmed by radiological and clinical investigation, respectively, were reported in the whole cohort (Figure 3).

## 4. Discussion

Nowadays, mi-TA represents the gold standard for uPHA over medical treatment [7]. Nonetheless, over the last decade, mi-PA has been increasingly adopted as an alternative surgical option for Conn’s syndrome, providing comparable outcomes with lesser incidence of cortisol replacement to mi-TA on the long run [19]. Despite this theoretical benefit, mi-PA remains currently underused due to lack of clear guidelines recommendation and granularity of available data [10]. Recently, Li et al. reported results of a pooled meta-analysis comparing PA vs. TA without providing a subgroup analysis accounting for different surgical techniques [20]. In this context, considering the growing interest towards robot-assisted adrenalectomy and the well-established role for laparoscopic adrenalectomy over years, we restricted our analysis to minimally-invasive adrenalectomy. Herein, we attempted to provide a more representative summary of current uPHA management among surgeons and urologists. 

Our study showed interesting findings. Compared to mi-TA, among eligible studies, mi-PA showed a significant trend over reduced mean operative time, despite being within a large retrospective cohort; Walz et al. reported no significant differences in terms of this variable when considering all adrenal benign masses amenable of surgical treatment [14]. Additionally, in our study, mi-PA patients showed both lower mean hospital stay (MD: −0.32 days; *p* = 0.004) and lower rate of overall postoperative complication relative to mi-TA (15.6 vs. 29.2%; *p* = 0.009). Interestingly, when adopting a more comprehensive definition of perioperative complication (CD ≥ 2), we found no differences between mi-PA vs. mi-TA (11.2 vs. 17.1%; *p* = 0.15). 

A major advantage of mi-PA over mi-TA may be represented by the decreased incidence of postoperative hypocortisolism. An increased rate of postoperative subclinical transient hypocortisolism after mi-TA was reported by Billman et al. (25.0% vs. 11.5% after mi-TA and mi-PA, respectively; *p* < 0.001) [36]. However, none of these patients needed permanent steroid replacement at median follow up of 24 months. This trend was also reported in prior series [11,34,35]. Conversely, a higher steroid replacement rate was reported by Anceschi et al., when compared mi-TA vs. mi-PA [10]. To date, available data suggest a negligible incidence of long-term steroid replacement after unilateral adrenalectomy for uPHA, irrespectively of the surgical approach (3–4.5%) [36]. 

Clinical success rate reported after surgery for uPHA remains controversial among series, ranging from 16% to 72% [38,39,40,41,42]. This wide variation highlights lack of consensus for defining clear success of adrenalectomy for PHA, irrespective of the surgical approach used [42]. In 2017, a collaborative international consortium (PASO) introduced a structured presentation of functional outcomes for adrenalectomy, establishing a comprehensive definition of complete, partial or absent clinical and biochemical success [27]. Several studies validated the greater accuracy of these new criteria in properly classifying postoperative functional results [41,42,43]. Nonetheless, Vorselaars et al. claimed that the use of high thresholds for definition of clinically relevant change in SBP, as well as the use of percentage, instead of absolute values, for evaluating daily-defined dose of medical treatment, represent the main drawbacks of these criteria [41]. To the best of our knowledge, only two published studies reported the comparison of clinical success between mi-PA and mi-TA according to PASO criteria [10,36]. We recorded comparable rate of complete, partial and absent clinical success between mi-PA and mi-TA according to PASO criteria (each *p* > 0.05). Interestingly, high rates of hypertension recovery (93.8% vs. 95.1%) with relevant postoperative SBP decrease (M.D. −39.44 vs. −43.39 mmHg, respectively) were recorded in mi-PA and mi-TA series, respectively. Since a relatively small decrease of SBP (≥10 mmHg) in HTN patients reduces by 13% the risk of all-cause mortality, these results support a theoretical benefit of both mi-TA and mi-PA in maintenance of SBP control, even if a complete clinical success may not be expectable [44,45]. 

Although the rate of absent clinical success was reported in two studies, data regarding its underlying etiology remain uncertain. For example, only three studies in the current SR investigated recurrence rate [11,34,36]. Overall, only two cases of recurrence in the mi-PA group were recorded. These findings reflect the low rate of recurrence after PA for Conn’s syndrome, as described in a recent meta-analysis (event rate 2%; 95% CI 1–5%) [18]. Moreover, Simforoosh et al. reported no cases of recurrence in seven patients treated by mi-PA for Conn’s syndrome at an extended follow-up (8 years) [33]. Furthermore, absence of malignant tumor at final pathological examination in all cases supports the use of mi-PA as a safe strategy for the treatment of patients with uPHA. Notably, a substantial number of uPHA patients treated with mi-PA vs. mi-TA harbored hyperplasia instead of APA (10% vs. 19%, respectively). Previous studies demonstrated that the presence of micronodules in the residual tissue after PA would expose patients to PHA recurrence [46,47]. Consequently, clinicians should carefully outweigh the benefit of adrenal sparing-surgery in this specific scenario. 

Finally, in the current study a small amount of adrenalectomy was performed by robotic approach (3%). However, an increasing body of literature has addressed its potential benefit in terms of perioperative outcomes [16,48]. The advantages of robotic vs. laparoscopy technique [49], including its ancillary technology, such as near-infrared fluorescence imaging, may allow surgeons to better identify and excise adrenal masses, thus promoting the use of adrenal-sparing surgery [17,50,51,52].

This study is not devoid of limitations. Heterogeneity among eligible studies in preoperative evaluation and outcomes assessment represent intrinsic biases. For instance, preoperative adrenal vein sampling (AVS) was not routinely performed in all patients. Consequently, patients experiencing no complete clinical success might have a concomitant, functionally active, micro-nodule in the contralateral gland [53]. Finally, retrospective design, as well a limited follow-up, still precludes definitive conclusion on the non-inferiority of mi-PA vs. mi-TA for all evaluated endpoints. Notwithstanding these limitations, current literature support mi-PA as a safe surgical treatment for uPHA with mid-term functional outcomes comparable to mi-TA.

## 5. Conclusions

Adrenal-sparing techniques may provide patients affected by PHA with increased reserve of functional parenchyma. However, current findings may not be generalizable out of tertiary referral centers. Further evidence supported by either prospective series or multicentric RCTs are still required to determine those patients that might benefit mostly from either mi-PA or mi-TA for uPHA.

## Figures and Tables

**Table 1:** Summary of published research: eligible studies testing perioperative and functional outcomes of mi-PA versus mi-TA.

Author	Year	Study Design	Institution	Patients	Time	Surgical Procedure mi-PA vs. mi-TA	Surgical Technique	Follow-Up (mo) mi-PA vs. mi-TA	Inclusion Criteria	Exclusion Criteria	Diagnostic Test	Outcome
**Anceschi et al.**	2020	Retrospective	Multiple	90	2011–2020	29 vs. 61	Robotic (24) Lap (85)	41 vs. 46	(a) Primary Hyperaldosteronism; (b) Single and unilateral adrenal mass	(a) Follow-up < 18 mo (b) Bilateral adenomas (c) Malignant histology	(a) CT and/or MRI (b) AVS °	Perioperative and Functional outcomes *
**Billmann et al.**	2020	Retrospective matched-cohorts	Multiple	249	2008–2018	81 vs. 168	Lap	22.8 vs. 24.8	(a) Unilateral Primary Aldosteronism (b) adrenal mass<6cm	(a) Follow-up < 60 mo (b) malignant histology (c) no data on extent of adrenal resection	(a) CT and/or MRI (b) AVS ° (c) Biochemical test	Perioperative and Functional outcomes *
**Liu et al.**	2020	Retrospective	Single	96	2012–2017	65 vs. 31	Lap	32.3 vs. 40.8	(a) APA	(a) IHA (b) Bilateral adenoma (c) UAH	(a) CT scan (b) Biochemical test	Perioperative and Functional outcome
**Chen et al.**	2014	Prospective	Single	63	2008–2011	16 vs. 47	Lap	12	(a) Primary Aldosteronism (b) Unilateral adrenal mass	NA	(a) CT scan (b) AVS ° or Scintigraphy (c) Biochemical test	Perioperative and Functional outcomes *
**Fu et al.**	2011	RCT	Single	212	2000–2004	104 vs. 108	Lap	96	(a) APA	(a) Previous ipsilateral adrenal surgery; (b) Doubtful BAH	(a) CT and/or MRI (b) AVS ° (c) Biochemical test	Perioperative and Functional outcome
**Ishidoya et al.**	2005	Retrospective	Single	92	1995–2004	29 vs. 63	Lap	60.3 vs. 29.3	(a) APA	(a) IHA	(a) CT scan (b) AVS ° (c) Biochemical test	Parioperative outcome
APA = aldosterone producing adenoma, IHA = idiopathic aldosteronism, UAH = unilateral adrenal hypeplasia, mo = months. * Use of PASO (Primary Aldosteronism Surgical Outcome study) criteria for the assessment of Clinical and/or Biochemical success. ° Adrenal venous sampling (AVS) was not performed in all patients and no clear indication were reported in most studies.												

**Table 2:** Summary of published research: Baseline characteristics among eligible studies testing perioperative and functional outcomes of PA vs. TA.

Author	Patients	Age	Gender (M:F)	Tumor Size (cm)	Side (R:L)	ASA (≤2:>2) or Mean (SD)	BMI	Surgical Approach (Rt:Tr)	HYT Duration (years)	ant-HYT Drugs (n°)	SBP (mmHg)	DBO (mmHg)	sP (mmol/L)	sA (ng/dL)	sRA (ng/mL/h)
**Anceschi et al.,** **2020**	PA (29)	median (IQR) 57 (43.5–67.5)	13:16	median (IQR) 2.7 (1.8–2.85)	7:22	23;6	NA	2:27	NA	NA	NA	NA	NA	NA	NA
	TA (61)	median (IQR) 54 (44.5–63)	23:38	median (IQR) 4.2 (2.35–6)	38:23	50;11	NA	20:41	NA	NA	NA	NA	NA	NA	NA
**Billmann et al.,** **2020**	PA (81)	mean (SD) 45.9 (14)	46:35	mean (SD) 1.8 (1.6)	36:45	80;1	mean (SD) 27 (4)	NA	median (range) 8 (2–12)	median (range) 3 (2–5)	NA	NA	NA	NA	NA
	TA (168)	mean (SD) 53.1 (15)	69:99	mean (SD) 3.4 (2.3)	81:87	158;8	mean (SD) 25 (5)	NA	median (range) 8 (2–13)	median (range) 3 (2–5)	NA	NA	NA	NA	NA
**Liu et al.,** **2020**	PA (65)	mean (SD) 48.27 (11.47)	22:43	mean (SD) 1.86 (0.65)	27:38	NA	NA	65:0	NA	NA	mean (SD) 166.52 (24.08)	mean (SD) 101 (14.12)	mean (SD) 2.55 (0.72)	mean (SD) 26.65 (6.89)	NA
	TA (31)	mean (SD) 56.42 (16.42)	12:19	mean (SD) 1.96 (0.96)	13:18	NA	NA	31:0	NA	NA	mean (SD) 174 (36.06)	mean (SD) 100.75 (21.44)	mean (SD) 2.37 (0.63)	mean (SD) 27.29 (7.60)	NA
**Chen et al.,** **2014**	PA (16)	mean (SD) 48.5 (10.9)	7:9	mean (SD) 2.02 (0.91)	11:5	NA	mean (SD) 23.3 (2.61)	0:16	NA	NA	mean (SD) 150.4 (26.9)	mean (SD) 92.1 (14)	mean (SD) 3.52 (0.63)	mean (SD) 57.3 (24.8)	mean (SD) 0.43 (0.82)
	TA (47)	mean (SD) 48.7 (11.3)	21:26	mean (SD) 2.10 (0.83)	25:22	NA	mean (SD) 23.3 (2.60)	0:47	NA	NA	mean (SD) 155.3 (23.5)	mean (SD) 93.7 (15.7)	mean (SD) 3.36 (0.76)	mean (SD) 54.8 (37.4)	mean (SD) 0.75 (1.89)
**Fu et al.,** **2011**	PA (104)	mean (SD) 43 (5.8)	45:59	mean (SD) 1.9 (0.2)	58:46	2.3 (1.7)	mean (SD) 26.2 (4.2)	104:0	mean (SD) 5.9 (1.2)	mean (SD) 2.2 (0.8)	mean (SD) 175.9 (23)	mean (SD) 101.8 (13)	mean (SD) 3 (3,4)	mean (SD) 34.5 (16)	mean (SD) 0.27 (0.4)
	TA (108)	mean (SD) 41 (7.8)	48:60	mean (SD) 1.8 (0.4)	55:53	2.6 (1.3)	mean (SD) 25.7 (3.5)	108:0	mean (SD) 6.3 (1.3)	mean (SD) 2.1 (0.8)	Mean (SD) 179.5 (24)	Mean (SD) 108.3 (14)	Mean (SD) 3.1 (0.5)	mean (SD) 32.2 (15)	mean (SD) 0.24 (0.6)
**Ishidoya et al.,** **2005**	PA (29)	median (range) 49.3 (30–68)	15:14	median (range) 1.55 (0.6–2.4)	17:12	NA	NA	23:6	NA	NA	NA	NA	NA	NA	NA
	TA (63)	median (range) 50.2 (29–75)	31:32	median (range) 1.6 (0.3–4)	25:37	NA	NA	0:63	NA	NA	NA	NA	NA	NA	NA
NA = not avialble; BMI = body mass index; HYT = hypertension; SBP = systolic blood pressure; DBO = diastolic blood pressure; sP = serum potassium, sA = serum aldosterone; sRA = serum Renin Activity.															

## Data Availability

No new data were created or analyzed in this study. Data sharing is not applicable to this article.

## Supplementary Materials

The following supporting information can be downloaded at https://www.mdpi.com/article/10.3390/jcm11051263/s1: Figure S1, Assessment of risk of bias according to ROBINS-I tool (a) and RoB 2 tool (b) as suggested by Cochrane handbook; Figure S2, Cumulative analysis of eligible studies comparing mi-PA vs. mi-TA in terms of (a) gender, (b) tumor side, (c) tumor size, (d) ASA 1-2, (e) ASA 3-4, (f) BMI, (g) surgical approach, (h) preoperative SPB, (i) preoperative DBP, (j) preoperative hypertension duration in years, (k) preoperative serum aldosterone, (l) preoperative renin activity and (m) serum potassium.
