# Supplementary material for: Minimally Invasive Partial vs. Total Adrenalectomy for the Treatment of Unilateral Primary Aldosteronism: A Systematic Review and Meta-Analysis
Source: J Clin Med. 2022 Feb 25;11(5):1263. doi: 10.3390/jcm11051263 (PMC8911420; doi:10.3390/jcm11051263)
Supplement: Supplementary file 1 [file jcm-11-01263-s001.zip › jcm-1551547-supplementary.pdf]

Supplementary Material

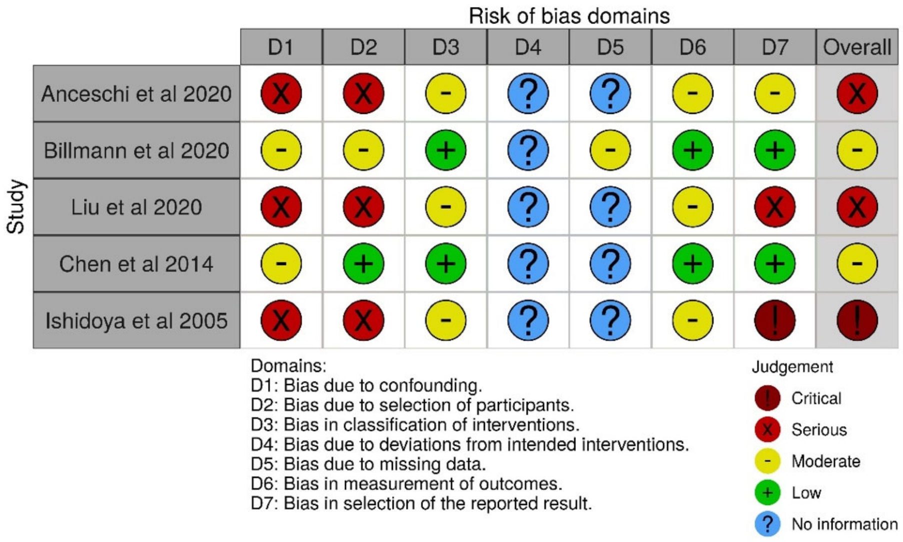

(**a**)

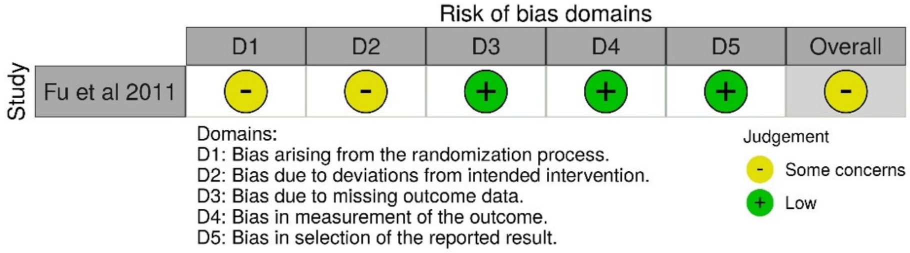

(**b**)

Figure S1. Assessment of risk of bias according to ROBINS-I tool (a) and RoB 2 tool (b) as suggested by Cochrane handbook

a) Forest plot representing analysis of **gender** (male vs female). CI = confidence interval; M-H = Mantel-Haenszel.

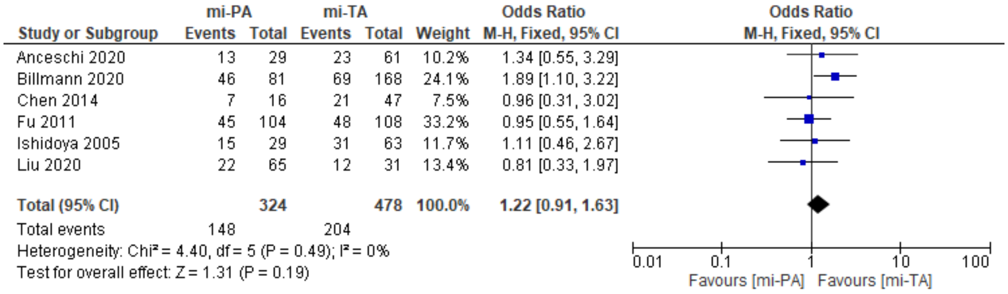

b) Forest plot representing analysis of **tumor side** (right vs left). CI = confidence interval; M-H = Mantel-Haenszel.

Study or Subgroup	mi-PA Events	mi-PA Total	mi-TA Events	mi-TA Total	Weight	Odds Ratio M-H, Fixed, 95% CI
Anceschi 2020	7	29	38	61	20.1%	0.19 [0.07, 0.52]
Billmann 2020	36	81	81	168	31.6%	0.86 [0.50, 1.46]
Chen 2014	11	16	25	47	4.3%	1.94 [0.58, 6.44]
Fu 2011	58	104	55	108	25.8%	1.22 [0.71, 2.09]
Ishidoya 2005	17	29	25	62	7.1%	2.10 [0.86, 5.14]
Liu 2020	27	65	13	31	11.1%	0.98 [0.41, 2.34]
**Total (95% CI)**		**324**		**477**	**100.0%**	**0.97 [0.72, 1.29]**
Total events	156		237			

Heterogeneity: Chi² = 15.11, df = 5 (P = 0.010); I² = 67%
Test for overall effect: Z = 0.24 (P = 0.81)

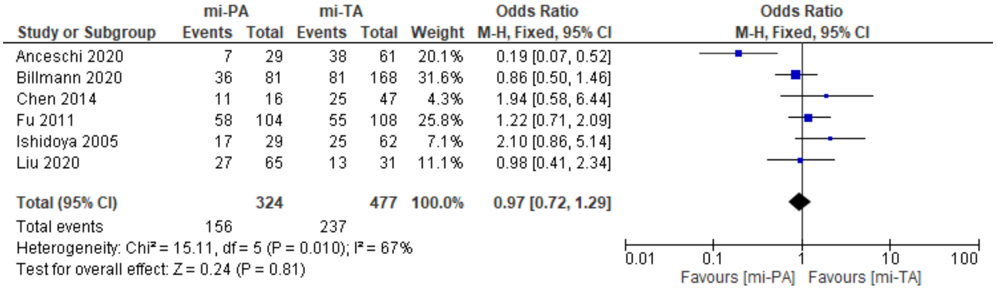

c) Forest plot representing analysis of **tumor size**. CI = confidence interval; M-H = Mantel-Haenszel.

Study or Subgroup	mi-PA Mean	mi-PA SD	mi-PA Total	mi-TA Mean	mi-TA SD	mi-TA Total	Weight	Mean Difference IV, Fixed, 95% CI
Anceschi 2020	2.41	0.56	29	4.12	2	61	2.0%	-1.71 [-2.25, -1.17]
Billmann 2020	1.8	1.6	81	3.4	2.3	168	2.4%	-1.60 [-2.09, -1.11]
Chen 2014	2.02	0.91	16	2.1	0.83	47	2.3%	-0.08 [-0.59, 0.43]
Fu 2011	1.9	0.2	104	1.8	0.4	108	80.9%	0.10 [0.02, 0.18]
Ishidoya 2005	1.53	0.46	29	1.7	0.83	63	8.3%	-0.17 [-0.43, 0.09]
Liu 2020	1.86	0.65	65	1.96	0.96	31	4.2%	-0.10 [-0.47, 0.27]
**Total (95% CI)**			**324**			**478**	**100.0%**	**-0.01 [-0.09, 0.06]**

Heterogeneity: Chi² = 86.09, df = 5 (P < 0.00001); I² = 94%
Test for overall effect: Z = 0.29 (P = 0.77)

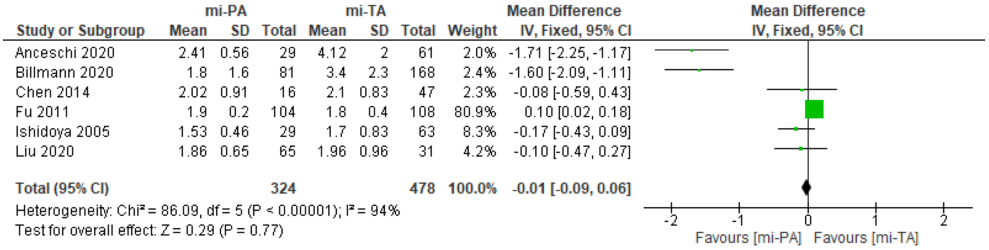

d) Forest plot representing analysis of **ASA 1-2**. CI = confidence interval; M-H = Mantel-Haenszel.

Study or Subgroup	mi-PA Events	mi-PA Total	mi-TA Events	mi-TA Total	Weight	Odds Ratio M-H, Fixed, 95% CI
Anceschi 2020	23	29	50	61	84.0%	0.84 [0.28, 2.56]
Billmann 2020	80	81	158	168	16.0%	5.06 [0.64, 40.25]
**Total (95% CI)**		**110**		**229**	**100.0%**	**1.52 [0.61, 3.81]**
Total events	103		208			

Heterogeneity: Chi² = 2.37, df = 1 (P = 0.12); I² = 58%
Test for overall effect: Z = 0.89 (P = 0.37)

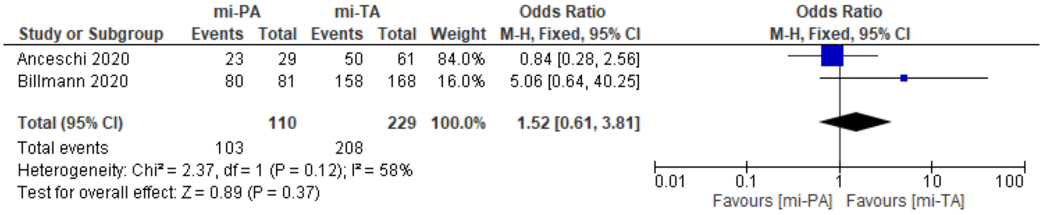

e) Forest plot representing analysis of **ASA 3-4**. CI = confidence interval; M-H = Mantel- Haenszel.

Study or Subgroup	mi-PA Events	mi-PA Total	mi-TA Events	mi-TA Total	Weight	Odds Ratio M-H, Fixed, 95% CI
Anceschi 2020	6	29	11	61	52.2%	1.19 [0.39, 3.60]
Billmann 2020	1	81	8	168	47.8%	0.25 [0.03, 2.03]
**Total (95% CI)**		**110**		**229**	**100.0%**	**0.74 [0.29, 1.88]**
Total events	7		19			

Heterogeneity: Chi² = 1.72, df = 1 (P = 0.19); I² = 42%
Test for overall effect: Z = 0.64 (P = 0.53)

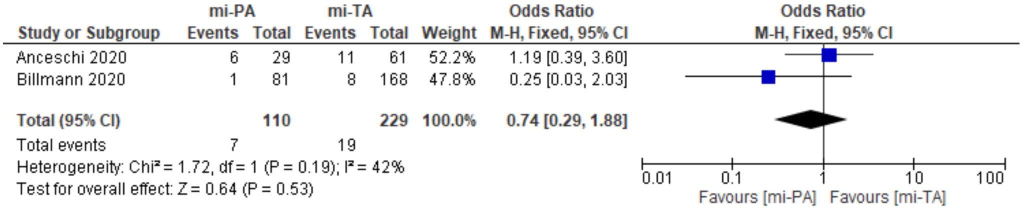

f) Forest plot representing analysis of **BMI** (Kg/m^2). CI = confidence interval; IV = inverse variance; SD = standard deviation.

Study or Subgroup	mi-PA Mean	mi-PA SD	mi-PA Total	mi-TA Mean	mi-TA SD	mi-TA Total	Weight	Mean Difference IV, Fixed, 95% CI
Billmann 2020	27	4	81	25	5	168	35.3%	2.00 [0.85, 3.15]
Chen 2014	23.3	2.61	16	23.3	2.6	47	21.5%	0.00 [-1.48, 1.48]
Fu 2011	26.2	4.2	104	25.7	3.5	108	43.2%	0.50 [-0.54, 1.54]
**Total (95% CI)**			**201**			**323**	**100.0%**	**0.92 [0.24, 1.61]**

Heterogeneity: Chi² = 5.48, df = 2 (P = 0.06); I² = 63%
Test for overall effect: Z = 2.64 (P = 0.008)

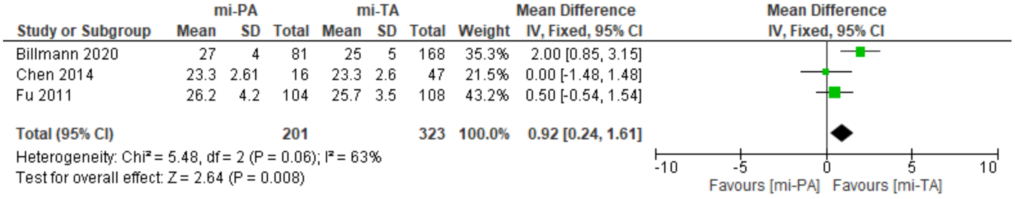

g) Forest plot representing analysis of **surgical approach** (retroperitoneal vs transperitoneal) . CI = confidence interval; M-H = Mantel-Haenszel.

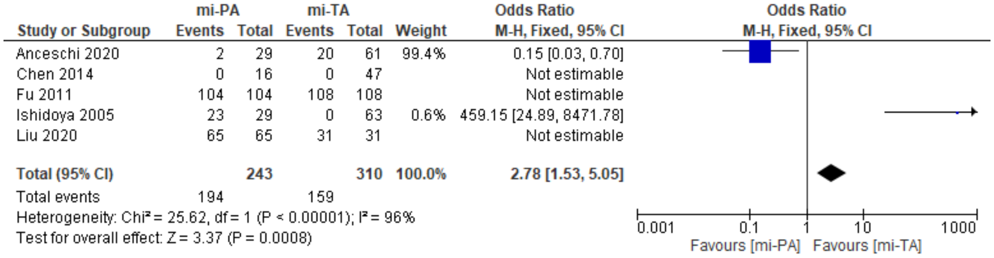

h) Forest plot representing analysis of **preoperative SPB** (mmHg). CI = confidence interval; IV = inverse variance; SD = standard deviation.

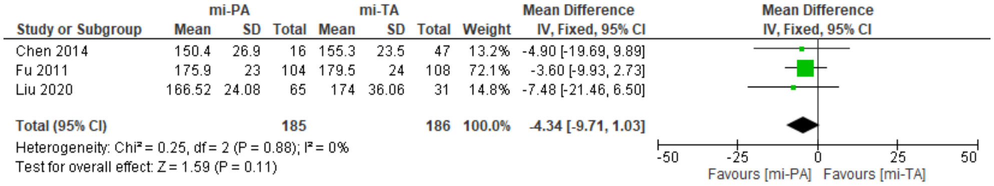

i) Forest plot representing analysis of **preoperative DPB** (mmHg). CI = confidence interval; IV = inverse variance; SD = standard deviation.

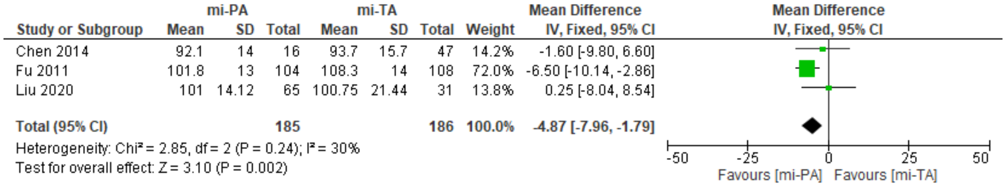

j) Forest plot representing analysis **preoperative HTN duration** (years). CI = confidence interval; IV = inverse variance; SD = standard deviation.

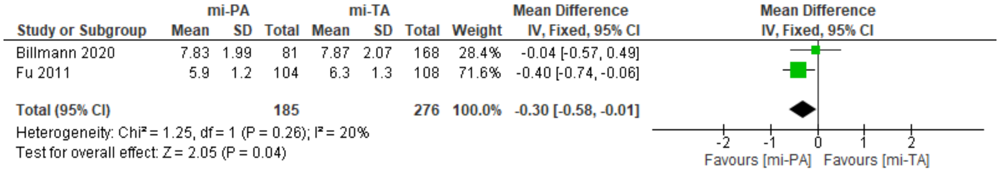

k) Forest plot representing analysis of **preoperative serum aldosterone** (ng/dl). CI = confidence interval; IV = inverse variance; SD = standard deviation.

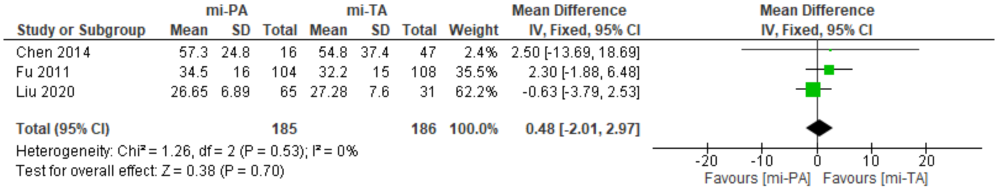

l) Forest plot representing analysis of **preoperative renin activity** (ng/ml/hr). CI = confidence interval; IV = inverse variance; SD = standard deviation.

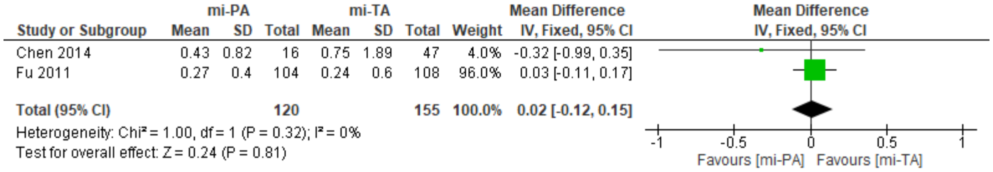

m) Forest plot representing analysis of **serum postassium** (mmol/l). CI = confidence interval; IV = inverse variance; SD = standard deviation.

	mi-PA			mi-TA				Mean Difference
Study or Subgroup	Mean	SD	Total	Mean	SD	Total	Weight	IV, Fixed, 95% CI
Chen 2014	3.52	0.63	16	3.36	0.76	47	32.1%	0.16 [-0.22, 0.54]
Fu 2011	3	3.4	104	3.1	0.5	108	10.5%	-0.10 [-0.76, 0.56]
Liu 2020	2.55	0.72	65	2.37	0.63	31	57.4%	0.18 [-0.10, 0.46]
**Total (95% CI)**			**185**			**186**	**100.0%**	**0.14 [-0.07, 0.36]**

Heterogeneity: Chi² = 0.59, df = 2 (P = 0.74); I² = 0%
Test for overall effect: Z = 1.32 (P = 0.19)

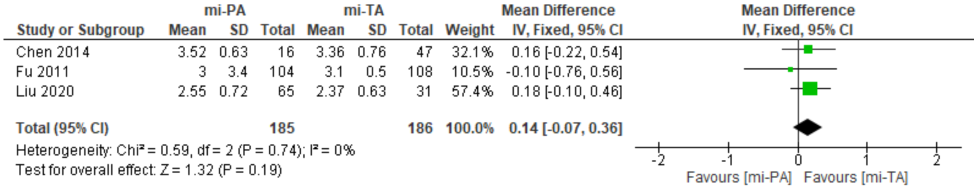

Figure S2. Cumulative analysis of eligible studies comparing mi-PA vs mi-TA in terms of (a) gender, (b) tumor side, (c) tumor size, (d) ASA 1-2, (e) ASA 3-4, (f) BMI, (g) surgical approach, (h) preoperative SPB, (i) preoperative DBP, (j) preoperative hypertension duration in years, (k) preoperative serum aldosterone, (l) preoperative renin activity and (m) serum postassium. Each studies is represented by a square incorporating confidence intervals represented by horizontal lines. The area of each square is proportional to the study's weight in the meta-analysis. The meta-analysed measure of effect is plotted as a diamond with lateral points indicating confidence intervals.
